# Ultrasound-Based Scaffold-Free Core-Shell Multicellular Tumor Spheroid Formation

**DOI:** 10.3390/mi12030329

**Published:** 2021-03-20

**Authors:** Karl Olofsson, Valentina Carannante, Madoka Takai, Björn Önfelt, Martin Wiklund

**Affiliations:** 1 Department of Applied Physics, Science for Life Laboratory, KTH Royal Institute of Technology, SE-106 91 Stockholm, Sweden; karlolo@kth.se (K.O.); onfelt@kth.se (B.Ö.); 2 Department of Microbiology, Tumor and Cell Biology, Science for Life Laboratory, Karolinska Institutet, SE-171 65 Stockholm, Sweden; valentina.carannante@ki.se; 3 Department of Bioengineering, University of Tokyo, 7-3-1 Hongo, Bunkyo-Ku, Tokyo 113-8656, Japan; takai@bis.t.u-tokyo.ac.jp

**Keywords:** multicellular tumor spheroids, core-shell spheroids, 3D image analysis, 3D culture, acoustophoresis, multiwell microplate

## Abstract

In cancer research and drug screening, multicellular tumor spheroids (MCTSs) are a popular model to bridge the gap between in vitro and in vivo. However, the current techniques to culture mixed co-culture MCTSs do not mimic the structural architecture and cellular spatial distribution in solid tumors. In this study we present an acoustic trapping-based core-shell MCTSs culture method using sequential seeding of the core and shell cells into microwells coated with a protein repellent coating. Scaffold-free core-shell ovarian cancer OVCAR-8 cell line MCTSs were cultured, stained, cleared and confocally imaged on-chip. Image analysis techniques were used to quantify the shell thickness (23.2 ± 1.8 µm) and shell coverage percentage (91.2 ± 2.8%). We also show that the shell thickness was evenly distributed over the MCTS cores with the exception of being slightly thinner close to the microwell bottom. This scaffold-free core-shell MCTSs formation technique and the analysis tools presented herein could be used as an internal migration assay within the MCTS or to form core-shell MCTS co-cultures to study therapy response or the interaction between tumor and stromal cells.

## 1. Introduction

Solid cancer tumors are cells embedded in a complex tissue architecture allowing cellular communication through direct contact or soluble factors [1,2]. Together with the tumor specific biophysical and biochemical factors permeating the 3D architecture, the tumor microenvironment influences cell behavior and tumor progression [3]. Standard in vitro 2D cultures, where cells are maintained on flat surfaces, do not mimic crucial aspects of the solid tumor and its microenvironment [4].

A popular tool for introducing higher complexity into in vitro cultures is the multicellular tumor spheroid (MCTS) which is a spherical aggregate of cells of a single or several tissue origins. The MCTS concept is appreciated because it integrates some of the advantages with standard 2D cultures, such as robustness and reproducibility, with biochemical and biophysical factors present in an in vivo solid tumor such as gas- and nutrient gradients and a spatial architecture that better supports cell–cell interactions [5,6]. In general, MCTS formation and culture techniques are either based on embedding cells in scaffolds or on scaffold-free aggregation of cells into a spherical shape [7]. Many of the scaffold MCTS formation methods utilize two-phase droplet generation within microfluidic channels or spontaneous cell aggregation in bulk scaffolds [8]. These techniques have the potential to form large quantities of uniformly sized MCTSs where the scaffold provides a mechanically controlled in vivo-like environment for cells to grow and organize. The challenges when using scaffold-based methods are batch-to-batch animal origin extra cellular matrix differences, low initial seeding density and difficulties in retrieving the cells for post-culture characterization [9]. The scaffold-free methods, relying on external forces for cell aggregation, take advantage of cell–cell interaction to create dense cell aggregates. Relying on cell–cell contact during MCTS formation greatly reduces time until the scaffold-free MCTSs are physiologically relevant models for solid tumors due to high initial cell density [10]. The most extensively used external force is gravity which in combination with low-adhesion surfaces or hanging drops forms MCTSs [11,12]. More active external forces, such as magnetic and centrifugal, manipulate cells into aggregates which mature into MCTSs [13,14].

Another useful external force is the acoustic radiation force which is a gentle and non-invasive way to manipulate the position of cells and beads [15]. The acoustic radiation force is experienced by a cell in an ultrasonic standing wave (USW). USWs are commonly induced in a resonator cavity, such as microfluidic channels and microwells, with a width corresponding to half the wavelength. The force scales with cell volume, actuation frequency and the acoustic contrast factor, which is the difference in mechanical properties between the cell and the suspension medium. The acoustic contrast factor also decides whether the cell will be pushed towards the pressure node or anti-node [16]. For a cell suspended in regular medium or PBS, the acoustic contrast factor is positive and the cell will be pushed to the pressure node [17]. The acoustic radiation force has been proven to be safe for cells and has been used to create MCTSs in several device configurations [18,19,20,21,22].

To further increase the complexity in the MCTS model, there is a growing interest in co-culturing different cells together, for example, cancer cells with different characteristics or cancer and stromal cells. For example, the incorporation of stromal cells, such as fibroblasts, into the MCTS cultures is usually done by mixing the two cell types at the MCTS formation stage, which results in a random spatial distribution of the stromal cells and cancer cells [23]. This approach doesn’t fully capture the in vivo situation in solid tumors where the fibroblast cells are usually found in the tumor periphery [24,25]. Another example is MCTS investigations based on patient-derived material where tumor biopsies were acquired from different parts of the tumor. A mixed co-culture MCTS of, for example, peripheral and central tumor cells does not mimic the architecture of the source tumor. An alternative to the mixed co-culture MCTS model is the more physiologically relevant core-shell MCTS, where cancer cells in the core are embedded inside a layer of cells of a different origin. The available techniques for core-shell MCTS formation are based on scaffolds. Using double-emulsion droplet methods, core-shell droplets can be created where the cancer cells containing core scaffolds are surrounded by fibroblasts embedded in the shell scaffolds [26]. Similar approaches have also been used to form organoids with spatial heterogeneity [27,28].

In this study, we use a USW-based approach to produce scaffold-free core-shell MCTSs from single cells suspended in regular medium. We previously presented an USW-based MCTS culture platform where 100 uniformly sized MCTSs could be cultured and post-culture processed in a multiwell microplate [29]. This platform can be used to culture MCTSs for up to at least 7 days with retained viability [21,30]. Using this platform in combination with a two-step cell seeding protocol of ovarian carcinoma cell line OVCAR-8, small MCTS cores were precultured for 24 h in the microwells before being acoustically embedded in cells destined for the shell structure. The core-shell MCTSs were stained and mounted in a refractive index matching solution (RIMS) to allow whole MCTS confocal imaging. The confocal z-stacks were analyzed using in-house developed image analysis methods to quantify the shell and core dimensions, shell coverage and number of cells in the shell and core respectively. Using our methodology, the average shell coverage percentage was 91.2 ± 2.8 % (mean ± standard deviation, n = 20) and the shell thickness was 23.2 ± 1.8 µm at a 2:1 shell to core cell seeding ratio.

The scaffold-free core-shell MCTS formation approach presented herein could be used in drug screening applications and become a valuable tool in studies focusing on, for example, tumor-stroma interaction.

## 2. Materials and Methods

### 2.1. Cell Culture and Staining

Cells from the OVCAR-8 ovarian cancer cell line were cultured in complete cell medium consisting of RPMI-1640 GlutaMAX (Thermo-Fischer Scientific, Waltham, MA, USA) supplemented with 10% heat inactivated fetal bovine serum (Thermo-Fischer Scientific), 1X MEM Non-essential amino acid solution and 25 mM HEPES (Thermo-Fischer Scientific). Cells were kept in T75 flasks and maintained at 37 °C in 5% CO_2_. Cells were maintained by splitting every second day.

Two fluorescent dyes were used to distinguish cells in the core from cells in the shell; cell-permeant Far Red DDAO-SE (Invitrogen, Carlsbad, CA, USA) for cells in the shell and post-fixation DAPI (Invitrogen) for all cells in the MCTSs. The shell cells were prepared for staining before seeding by washing in PBS and centrifuging (300× *g* for 5 min) two times. The supernatant was discarded and the cells were incubated at 37 °C for 10 min in PBS with 5 µM Far Red DDAO-SE. Two centrifugal washing steps with complete cell medium were performed before the stained cells were resuspended to a final concentration of 4 × 10^5^ cells mL^−1^.

Post-fixation staining of the MCTSs was performed after 48 h in culture by washing 3 times in PBS before fixation in BD Cytofix/Cytoperm^TM^ solution (BD Biosciences, San Jose, CA, USA), containing 4.2% paraformaldehyde, for 10 min and protected from light. The fixed MCTSs were washed another 3 times in PBS before three 5-min washes with a wash buffer (2% bovine serum albumin (BSA) and 0.2% Triton X-100 in PBS) to prepare the MCTSs for staining. The MCTSs were incubated in a stain solution (0.1% BSA and 0.2% Triton X-100 in PBS) with 5 µg mL^−1^ DAPI (Invitrogen) for 1 h. The staining was followed by three 20-min washes using wash buffer before proceeding to the clearing step. The fixation and post-fixation staining steps were performed at room temperature and on-chip with the MCTSs retained in the microwells.

### 2.2. Ultrasound-Based MCTS Culture Platform

**Figure 1:**
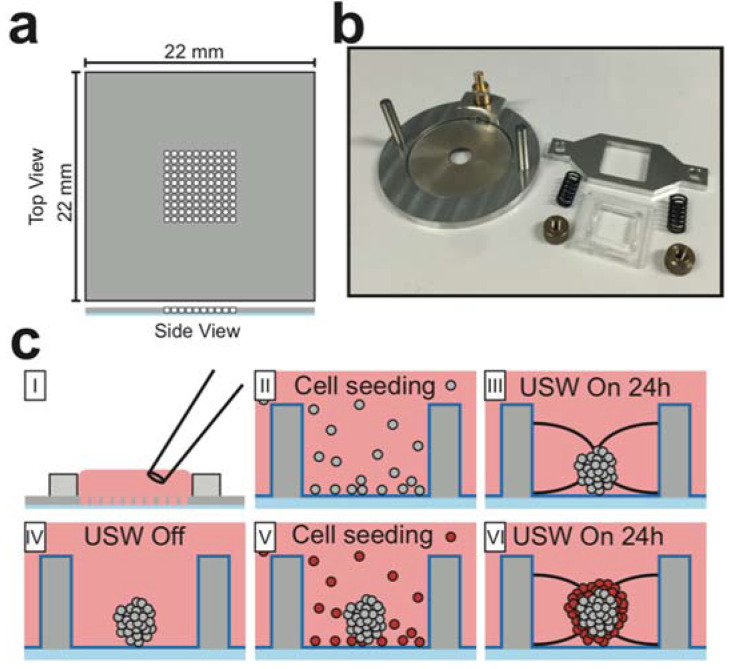
Ultrasound based layered multicellular tumor spheroids (MCTS) formation. The multi-well microplate (**a**) consists of a 22 × 22 × 0.3 mm^3^ silicon plate with 100 microwells (350 × 350 µm^2^) etched straight through the silicon layer and a 170 µm thick glass plate bonded to the bottom. The multiwell microplate was fixed on a circular transducer (**b**) with immersion oil as coupling medium to introduce ultrasonic standing waves (USWs) into the microwells. To form core-shell MCTSs (**c**), cells were seeded with a regular pipette into the shared medium reservoir above the microwells (I) and allowed to sediment to the well bottoms (II). The microplate was then transferred to the transducer which was actuated with a frequency corresponding to the λ/2-criterion across the microwell width. The radiation forces trapped all cells in an aggregate in the microwell center for 24 h (III). The microplate was dissembled from the transducer (IV) and cells comprising the shell were seeded around the preformed MCTS core (V) before the radiation forces were reintroduced for 24 h to create the core-shell MCTSs (VI).

The USW MCTS culture platform consists of a silicon-glass multiwell chip and a transducer fitted with chip clamping equipment which has previously been described in detail [21,29]. In short, the multiwell chip (Figure 1a) was produced by dry-etching 100 square holes (350 × 350 µm^2^) with concave walls through a 300 µm thick silicon wafer before being bonded to a 170 µm glass wafer and diced into a 22 × 22 mm^2^ chips [31]. The microwell dimensions sets an upper limit to the maximum MCTS diameter (350 µm) which is well below the limit for creating MCTSs with a necrotic core (450–500 µm) [32]. A PDMS gasket, bonded around the microwell array, provided a shared medium reservoir above the wells and liquid manipulation was done with a regular pipette. The crucial chip design feature is the glass bottom with a thickness corresponding to a No. 1.5 coverslip which gives excellent imaging properties. Cell attachment to the glass bottom and silicon walls was prevented by a protein repellent polymer-coating which was stabilized at the surfaces through a combination of covalent bonding and hydrophobic interaction within the coating layer [33]. The polymer coating was composed of 2-methcryloxypropyl phosphorylcholine (MPC), 3-methacryloxypropyl trimethoxysilane (MPTMSi) and 3-(methacryloyloxy)propyl-tris(trimethylsilyloxy) silane (MPTSSi). The poly (MPC-co-MTPMSi-co-MTPSSi) was used at 0.10 wt% in methanol during the coating step.

The transducer (Figure 1b) was comprised of an aluminum frame fitted with a circular piezo ceramic plate coupled with soldered wires to an SMB connector. The multiwell chip was placed on the piezo ceramic plate with immersion oil as coupling medium and retained in position with a plastic and aluminum frame fixed with springs and nuts.

### 2.3. Core-Shell MCTS Formation

Forming core-shell MCTSs was a two-step process where the MCTS core was aggregated for 24 h before adding the Far Red-stained cells comprising the shell (Figure 1c). To form the MCTS core, a single cell suspension (100 µL, 200,000 cells/ mL) was seeded with a pipette into the medium reservoir and the cells were allowed to sediment into the microwells. When the cells had settled at the microwell bottoms, a cover glass was placed over the shared medium reservoir to protect it from contamination. The microwell chip was placed in the transducer platform which was actuated with a frequency modulation scheme (2.47 ± 0.05 MHz, 1 kHz sweep rate) which corresponded to a half-wavelength resonance condition in the microwells. The actuation voltage was 15 Vpp over the piezo electrodes. The USW exerts acoustic radiation forces on the cells and traps them into aggregates in the pressure nodes located in the center of each well. The aggregates were stabilized and formed into small MCTS cores during 24 h with the USW turned on (active USW culture). After 24 h, the multiwell chip was disassembled from the transducer to seed the shell cells. The Far Red-stained cells destined for the MCTS shell were seeded (100 µL, 400,000 cells/mL) and the ultrasound procedure was repeated to trap the cells onto the preformed MCTS cores. The time between the second cell seeding step and USW start was kept as short as possible to avoid all the cells reaching the microwell bottom and enable the cells to be forced evenly over the core. The stochastic cell seeding into the microwells might generate a slight difference in the MCTSs volumetric properties.

### 2.4. MCTS Clearing, Microscopy and Imaging

To overcome the light scatter issues when imaging the MCTSs, a clearing protocol based on a refractive index matching solution (RIMS) was employed to render the MCTSs optically transparent. The ready stained MCTSs were mounted in RIMS, which for this study was 755 mg mL^−1^ Iohexol (Omnipaque 355 mg mL^−1^ Iodine, GE Healthcare, Chicago, IL, USA). To avoid the MCTSs escaping the microwells due to the comparably high RIMS density and to limit MCTS size changes due to rapid internal liquid exchange, the RIMS was introduced in the steps (10, 25, 50, 75 and 100% (RIMS/PBS vol/vol)).

Laser scanning confocal imaging (Zeiss LSM 880, Jena, Germany) was performed with the microwell chip mounted in a custom-built holder using a 20× objective. The pixel resolution was set to 512 × 512, with the pixel dimension 0.4393 × 0.4393 µm^2^ in combination with a pinhole size corresponding to 0.9 µm thick optical sections. These pixel dimensions were chosen to enable interpolation along the z-axis and to generate isotropic voxel size in the segmentation and image analysis stage. Laser intensity and image acquisition parameters were adjusted to avoid overexposed pixels throughout the MCTS volume. Optical sections were acquired with 0.9 µm section steps and the z-stacks covered the full MCTS volume.

### 2.5. Image Analysis and Post-Processing

**Figure 2:**
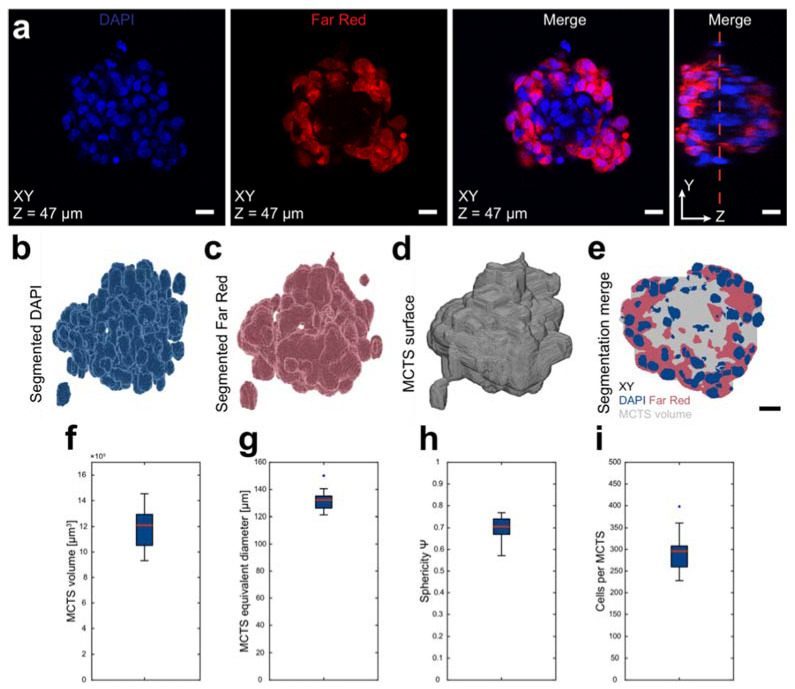
Raw data, segmentation strategy and core-shell MCTS characteristics. After 48 h (24 + 24 h) of USW formation, the core-shell MCTS were fixed, stained with DAPI (Invitrogen) and cleared. All cells were stained with DAPI while cells in the shell were identified by Far Red staining (**a**)**.** The red dotted line in the YZ plane indicates the XY optical section. Using a local adaptive thresholding algorithm, the DAPI (**b**) and Far Red (**c**) were segmented and used to approximate the MCTS surface and full volume (**d**). An XY-section in the merged segmented volume is shown as reference (**e**). The MCTS surfaces from 20 MCTSs imaged by confocal microscopy were used to measure layered MCTS volume (**f**), which has an equivalent diameter of a sphere with the same volume as the MCTS (**g**) and sphericity Ψ (**h**). Using the median nucleus volume after a watershed algorithm in the segmented DAPI, the number of cells was estimated as total DAPI volume divided by the volume of a single nucleus (**i**). Boxplots show the 25th and 75th percentiles with a red line marking the median value. The whiskers show the furthest observation 1.5 times the interquartile length away from the box edge while outliers are marked with a blue dot. Scalebars are 20 µm.

Confocal image z-stacks of DAPI and Far Red stained and cleared core-shell MCTSs (Figure 2a) were loaded into MATLAB-scripts that segmented the nuclei and the Far Red positive shell cells. All the operations were done in 3D and not sequentially on each individual optical section.

To prepare the raw data for initial segmentation, both the DAPI and Far Red channels in the image stack were interpolated in the z-direction to achieve an approximately isotropic voxel size (0.4393 × 0.4393 × 0.45 µm^3^) before a Gaussian filter was applied to smooth the image volume. A local adaptive threshold algorithm segmented the background and foreground based on local voxel mean intensity in a neighborhood volume corresponding to an eighth of the interpolated stack dimensions. In the segmented DAPI channel, a set of morphological operations and volume filtering on the thresholded volumes closed all the holes and removed small objects.

A seed-based watershed algorithm was used in steps on the segmented DAPI stack to separate nuclei clusters. The first set of seeds were generated by finding regional minima after applying a distance and H-minima transformation to the thresholded DAPI volume. After imposing the seeds as catchment basins in the distance transformed image stack, the watershed algorithm separated objects based on the seeds. To limit over segmentation, a second watershed was applied after removing seeds generating objects smaller than 70% of the median object volume.

MCTS morphological data, such as volume, surface area and equivalent diameter, were retrieved from the built-in MATLAB function regionprops3 and the sphericity Ψ was calculated as

$$\Psi =\frac{\pi^{\frac{1}{3}}{\left(6V_{\mathrm{MCTS}}\right)}^{\frac{2}{3}}}{A_{\mathrm{MCTS}}}$$

where *V*_MCTS_ is the MCTS volume and *A*_MCTS_ is the MCTS surface area [34]. The number of cells was approximated by dividing the total segmented DAPI volume by the median volume of a single nucleus [35]. This approach has been benchmarked with good agreement against manually counted cells in confocal z-stacks of DAPI stained MCTSs. The ratio of Far Red positive voxels was calculated by dividing the number of positive voxels to the total sum of voxels present in the volume of interest. Only voxels that were part of the whole MCTS volume were used. The segmentation and post-analysis were performed on a mid 2015 15-inch MacBook Pro.

## 3. Results

### 3.1. Layered MCTS Characteristics

After formation, the core-shell MCTSs were stained, and RIMS cleared and imaged (n = 20). The two-step seeding strategy for core-shell MCTS formation was investigated using two fluorophores; DAPI and FAR Red DDAO-SE. Since the DAPI staining was performed after fixation and permeabilization, all nuclei were DAPI positive while the shell was distinguished by the pre-seeding stained Far Red positive cells. The RIMS clearing strategy allowed for whole MCTS confocal imaging (Figure 2a).

The segmented DAPI (Figure 2b) and Far Red (Figure 2c) masks were used to approximate the full MCTS volume. The DAPI and Far Red segmented voxels were combined into a binary image volume and a dilatation and subsequent erosion operation were used to connect all voxels into a solid volume (Figure 2d). The segmented DAPI, Far Red and whole MCTS volumes were used in all subsequent analysis (Figure 2e). The MCTS volumes were used to investigate the volumetric parameters where the MCTS volume was 12.1 × 10^5^ ± 2.0 × 10^5^ µm^3^ (mean ± standard deviation, n = 20) (Figure 2f). The MCTS diameter was approximated by the diameter of a sphere with an equal volume and was 285.9 ± 15.3 µm (Figure 2g). The surface area and volume were used to calculate the sphericity ψ, a number between 0 and 1 where a perfect sphere has sphericity 1, and was found to be 0.70 ± 0.06 (Figure 2h). To estimate the number of cells in each MCTS, the total DAPI segmented volume was divided by the volume of a single nucleus and the MCTSs contained 293 ± 43 cells (Figure 2i). The single nucleus volume was found by taking the median nucleus volume after the watershed separation (Supplementary Figure S1).

### 3.2. Far Red Positive OVCAR8 Distribution

**Figure 3:**
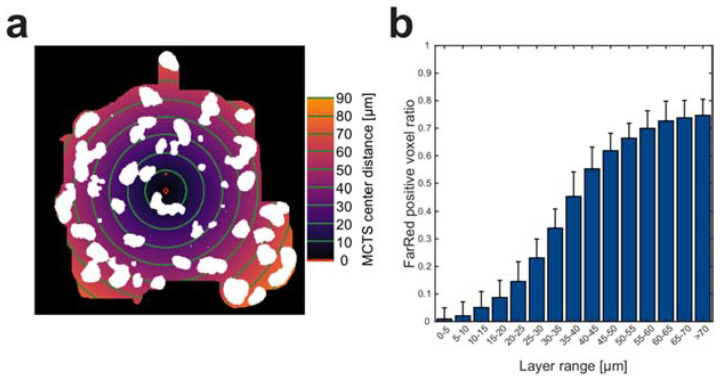
Cellular arrangement in core-shell spheroids. Each MCTS (n = 20) was divided into 5 µm thick concentric layers (every 10 µm marked by green lines) based on distance (colormap indicates distance away from center) from the MCTS center (red dot) (**a**). The ratio between Far Red positive voxels against total number of voxels was calculated in all layers (**b**). Scale bar is 20 µm.

The spatial distribution of Far Red positive voxels was measured by dividing the MCTSs into concentric layers around the MCTS core center points. The core center points were found by subtracting the segmented DAPI masks by the Far Red masks and finding the DAPI intensity weighted center of mass points in the MCTS volumes. A distance transform was used to calculate the distance from the core center points to each voxel within the MCTSs and 5 µm wide concentric layers were defined (Figure 3a). In each layer, the ratio of Far Red positive voxels was calculated (Figure 3b). Close to the MCTS center points (0–10 µm) there are very few Far Red positive voxels, while the ratio reaches around 0.7 in the shell 50–70 µm.

### 3.3. Shell Thickness and Coverage

**Figure 4:**
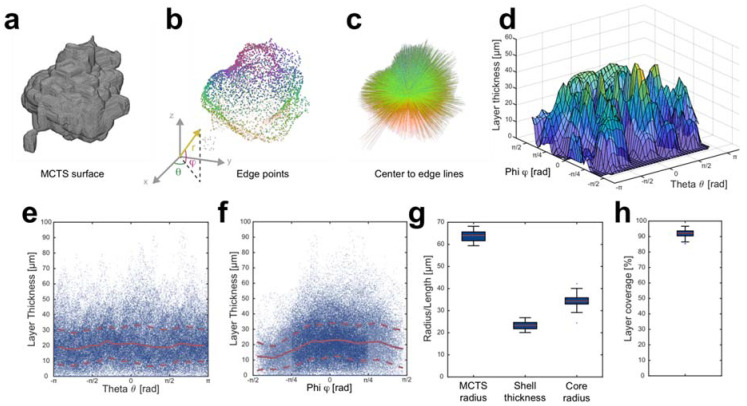
Center to edge line-analysis assessing direction dependent MCTS core and shell thickness distribution. Using the core-shell MCTS surface (**a**), 5000 equidistantly spaced points were defined on each MCTS by using spherical coordinates (radial distance *r*, polar angle *φ* [−π/2 ≤ *φ* ≤ π/2] and azimuth angle *θ* [−π ≤ *θ* ≤ π]) with the origin placed in the MCTS core center point (**b**). The voxels along the lines between the MCTS core center and each edge point (**c**) were evaluated in the segmented Far Red volume to acquire the shell thickness (number of Far Red positive voxels times length per voxel) as a function of the polar and azimuth angle in each MCTS (**d**). The color of the points and lines corresponds to the position along the *z*-axis (**b**,**c**). Line data points (blue dots) pooled from all (n = 20) core-shell MCTSs shows the shell thickness distribution as a function of azimuth (**e**) and polar angle (**f**). The mean (solid red line) and standard deviation (dashed red line) were calculated in π/10 (**e**) and π/20 (**f**) wide angle segments for the azimuth and polar angle respectively. The line data were used to measure the average MCTS radius, shell thickness and inner core radius in each MCTS (**g**). All lines not including any Far Red positive voxels were used to measure the shell core coverage percentage (**h**). Boxplots show the 25th and 75th percentiles with a red line marking the median value. The whiskers show the furthest observation 1.5 times the interquartile length away from the box edge while outliers are marked with a blue dot.

The spatial distribution of Far Red positive volume derived from the concentric layer approach does not provide full information of shell coverage, core radius and shell thickness. To study these characteristics in more detail, we developed a novel MCTS analysis strategy based on measuring voxel intensities along lines from the MCTS core center point to the surface. The core center point within the MCTS surface (Figure 4a) was used as origin when switching from Cartesian coordinates (*x, y, z*) to spherical coordinates (radial distance *r*, polar angle *φ* [−π/2 ≤ *φ* ≤ π/2] and azimuth angle *θ* [−π ≤ *θ* ≤ π]). The radial distance *r* coordinate is the distance between the center point and a surface point, the polar angle *φ* maps the height along the *z*-axis and the azimuth angle *θ* defines the surface point to the *x*- and *y*-axis. The advantages with using spherical coordinates when analyzing MCTSs are that it is well suited to the MCTS geometry and provides a tool to resolve direction dependent differences based on the angle coordinates. For each MCTS (n = 20), 5000 equally spaced points were defined on the surface (Figure 4b); the voxel indices creating a straight line from the center point to the surface points were calculated using Bresenham’s algorithm in 3D [36,37]. The line voxel indices can then be applied to any raw image channel or segmented volume to acquire image data. The data within the line indices can thus be viewed as a line histogram from the center to the surface (Supplementary Figure S2). The shell thickness was measured as the number of Far Red positive voxels along each line multiplied by the direction corrected voxel length and measured as a function of polar and azimuth angle for each MCTS (Figure 4d).

To investigate any systematic direction dependent differences in the shell thickness, the shell thickness distribution in 20 MCTSs was studied as a function of azimuth angle *θ* (Figure 4e) and polar angle *φ* (Figure 4f). The trend lines were defined as the mean shell thickness in π/10 (azimuth angle) and π/20 (polar angle) wide segments (solid red line ± standard deviation in dashed red lines). The azimuth angle segments include all shell thickness data in an arch from the MCTS top (*φ* = π/2) to bottom (*φ* = −π/2) and the polar angle segments include all thickness data along a cone surface with the circular opening ([−π ≤ *θ* ≤ π]) at different heights in the MCTS. The azimuth angle trend line indicates a relatively uniform shell thickness around 20 µm as a function of azimuth angle *θ*. The trend line for the shell thickness as a function of the polar angle *φ* shows that the shell was on average thinner (~12 µm) between −π/2 and −π/4 compared to the thickness above the −π/4 angle. The −π/2 to −π/4 polar angle region corresponds the bottom part of the MCTSs close to the microwell bottoms.

Overall, the MCTS radius, defined as the radial distance *r* between the central point and the surface points, was 64.0 ± 3.2 µm (mean ± standard deviation) while the shell thickness was 23.2 ± 1.8 µm (Figure 4g). The MCTS core radius, defined as the number of Far Red negative voxels before the first Far Red positive voxel multiplied by the direction corrected voxel length (Supplementary Figure S3), was found to be 34.3 ± 3.7 µm.

Another important metric for the core-shell MCTS formation is how well the shell covers the MCTS core. We used our center to surface line data to calculate the shell coverage percentage as the number of lines containing Far Red positive voxels divided by the total number of lines. Some of the lines did not include any Far Red positive voxels. The shell coverage percentage was 91.2 ± 2.8% (mean ± standard deviation) (Figure 4h).

### 3.4. Core and Shell Content

**Figure 5:**
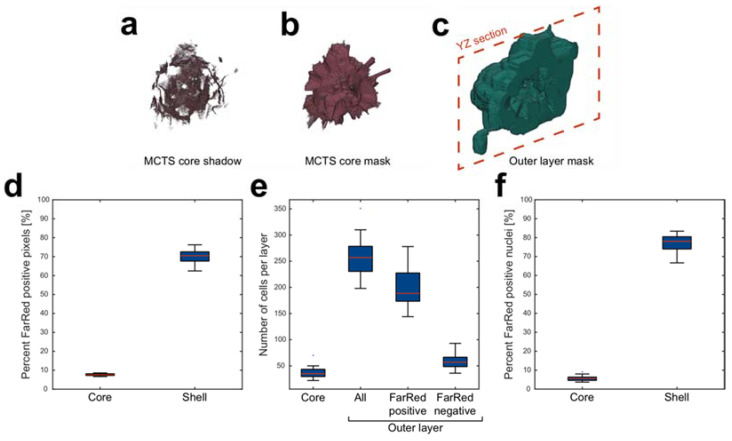
Edge-line analysis enables core and shell segmentation for content analysis. The outer edge points of the MCTS core (MCTS core shadow) was detected by identifying the first Far Red positive voxel in each edge-center line (**a**) and used to create a core mask (**b**). The full MCTS volume subtracted by the core mask was used to define the shell mask (centrally placed void illustrates the border between shell and core in the YZ plane marked by a dashed red line) (**c**). The ratio between far red positive voxels and the total amount of voxels was calculated (**d**) and the numbers of cells in the core, shell, far red positive and far red negative parts of the shell were estimated by dividing the total DAPI volume by the median nucleus volume in each compartment (**e**). The number of cells in each compartment was used to calculate the number of cells that was found in the Far Red positive volume (**f**). Boxplots show the 25th and 75th percentiles with a red line marking the median value. The whiskers show the furthest observation 1.5 times the interquartile length away from the box edge while outliers are marked with a blue dot.

With the center to surface line analysis outlined above, we could also quantify the content within the MCTS core and shell. In order to do so, the MCTS core and shell have to be compartmentalized into masks for subsequent analysis. We took advantage of the outer edge of MCTS core acquired from the indices before the first Far Red positive voxel in the line data to create the MCTS core “shadows” (Figure 5a). Expansive dilation and erosion morphological operations closed the structure into a core mask (Figure 5b) and the shell mask was acquired by subtracting the core mask from the full MCTS volume mask (Figure 5c). The ratios of Far Red positive voxels were calculated as the number of Far Red positive voxels divided by the total amount of voxels in cores and the shells from 20 core-shell MCTSs. The ratio was 7.6 ± 0.6% (mean ± standard deviation) Far Red positive voxels in the MCTS core and 70.2 ± 3.6% in the shell (Figure 5d).

The numbers of cells in the MCTS core and shell were estimated using the same strategy outlined above; the total DAPI positive volume divided by the median volume of a single nucleus. The MCTS cores contained 37.1 ± 11.1 (mean ± standard deviation) nuclei while the shell contained 255 ± 38.3 nuclei (Figure 5e). Since approximately 30% of the shell volume was Far Red negative, we also calculated the numbers of nuclei within the Far Red positive and Far Red negative shell volume. The number of nuclei within the Far Red positive shell volume was 197.0 ± 33.0 (mean ± standard deviation) while 58.1 ± 13.5 nuclei was found in the Far Red negative shell volume. When combining the cell numbers in the core and shell, the numbers of nuclei in the positive (198.9 ± 33.4 cells) and negative (93.5 ± 17.7 cells) Far Red volumes correspond well to the 2:1 seeding ratio. The numbers of cells in the core and shell were also used to complement the voxel ratio data (Figure 5d) by estimating the ratio of Far Red positive cells in the core and shell compartments (Figure 5f). It was found that 5.7 ± 1.4% and 77.2 ± 4.5% of the cells were Far Red positive in the core and shell respectively.

## 4. Discussion

We have demonstrated a USW-based technique for parallel formation and culture of a 100 uniformly sized scaffold-free core-shell MCTSs in a multiwell microplate. Using in-house developed image analysis scripts, the formed core-shell MCTSs were characterized and the core and shell dimensions were quantified in detail. The spatial arrangement of cells could be useful to augment or better model solid tumors and organoids. For example, scaffold-based organoid droplets with a HepG2 core and NIH-3T3 fibroblast shell were shown to have increased liver specific functions, such as urea and albumin synthesis, compared to spheroids only containing HepG2 [27]. Another scaffold-based droplet study showed that an MCF-7 core enclosed in a fibroblast shell was more resistant to the drugs paclitaxel and curcumin compared to fibroblast and MCF-7 mixed co-culture MCTSs [26].

The center to surface line analysis we developed and applied to the core-shell MCTSs enabled detailed characterization and quantification of several parameters. Using spherical coordinates with the origin in the MCTS center enabled the investigation of Far Red positive voxels as a function of the polar and azimuth angle. The shell thickness did not depend on the azimuth angle *θ* while the shell thickness was lower in the −π/2 to −π/4 polar angle region, which corresponds to the lower parts of the MCTSs. Since the preformed MCTS cores rest in microwell bottoms and we use a 2D horizontally oriented acoustic standing wave to trap the shell cells onto the core, the lower part is expected to have a lower shell thickness. The combination of acoustic radiation forces and possibly acoustic streaming were in tandem able to on average cover the bottom part of the MCTSs without any active levitation of the spheroids. The acoustic streaming in our platform has previously been characterized, albeit at higher actuation voltages compared to the voltages used in this study [38]. We hypothesize that the acoustic streaming around the MCTSs plays an important role for achieving a uniform shell thickness. In addition, the protein repellent coating was also important to prevent the MCTS core from adhering to the microwell bottom. The coating has excellent cell and bacteria repellent properties while the coating thickness at the concentration used does not interfere with the USW [29,39]. The line data analysis also showed a coverage percentage of 91.2 ± 2.8% (mean ± standard deviation, n = 20).

Another quantification that was enabled by the center to surface line analysis was the nuclei content and percentage of Far Red positive voxels in the core and the shell. Using the line data, we were able to create a mask for the core and the shell which we used to measure the contents. This was especially valuable when comparing the Far Red positive percentage using the quantification based on distance (Figure 3b) and the core-shell mask analysis (Figure 5d). The Far Red positive voxel percentages in the distance range 0–10 µm and 50–70 µm away from the MCTS center correspond very well to the results from the core-shell mask analysis. The smooth Far Red positive voxel percentage transition between the inner and outer distances can thus be explained by an off-center and non-spherical MCTS core and not by a floating border between the shell and core. The core-shell mask content analysis instead shows a distinct border between the two compartments after 24 h of culture using our ultrasound-based method. The distinct border after 24 h of core-shell culture might change in prolonged single cell type core-shell MCTS cultures or co-culture core-shell MCTSs.

The Far Red voxel percentage 70.2 ± 3.6% (mean ± standard deviation, n = 20) in the shell indicates though that there were some cells escaping the core and mixed with the shell. This mixing was also quantified by the percentage of cells that were Far Red positive (77.2 ± 4.5%). The discrepancy between the percentage of Far Red voxels and nuclei can be explained by the presence of non-Far Red voxels between the cells. The difference is unlikely to depend on a cell volume difference between the core and shell cells since they were from the same cell line. When calculating the numbers of nuclei in the MCTS core and shell, we found that the total numbers of nuclei in the Far Red negative and positive volumes corresponded well with the seeding ratio which was 1:2 (core:shell). While not in focus in this study, the seeding ratio between core and shell cells can easily be adjusted to control the core and shell dimensions. We have previously shown that the method to calculate the number of nuclei is accurate when using an image stack with small distances between the optical sections [35]. The degree of mixing between the shell and core is probably cell line dependent and could be used as an internal migration assay within the core-shell MCTSs, knowing the initial position of the shell cells and using the tools outlined in this study.

The insight provided by the surface to line analysis approach presented herein shows the potential to use this strategy with other applications. It is a facile way to get direction dependent information in spherical objects and can be applied to z-stacks of any MCTSs and droplets which is not straight forward using Cartesian coordinates (*x*,*y*,*z*). We think this analysis strategy will find good use in MCTS drug penetration investigations and immune cell infiltration assays.

While we only used a single cell line to illustrate and evaluate the USW-based core-shell MCTS formation method, other cell lines can easily be incorporated since the USW-based 3D culture method is very effective with any tested cell line [21,29]. Our study shows that acoustic radiation forces can be used to create scaffold-free core/shell MCTSs and the sequential seeding protocol presented herein could in principle be used in other USW-based devices as well. USW-based cell aggregation has been shown in layered resonators, surface acoustic wave devices and used in acoustic streaming [19,20,40,41,42,43]. The main advantages of using our multiwell microplate for MCTS formation and culture are the open system which allows for fluid handling using standard pipettes and that the microwells provide separate compartments for the MCTSs which enables long term surveillance and characterization. The challenge with using microwells as resonator chambers is the complex pressure node patterns which we have addressed by implementing a frequency modulation scheme [30].

## 5. Conclusions

We have demonstrated a scaffold-free core-shell MCTS formation and characterization platform using ultrasonic standing waves (USWs) in microwells. The core-shell MCTSs formed had a uniform shell layer thickness and a distinct border between the core and the shell. We believe that this method could be used in organoid and drug screening investigations where a higher degree of spatial control is required.

## Supplementary Materials

The following are available online at https://www.mdpi.com/2072-666X/12/3/329/s1, Figure S1: Median nucleus volume acquisition. Histogram shows the nucleus volume after watershed segmentation. The red dashed line indicates the median nucleus volume used to calculate the number of nuclei, Figure S2: Center to surface line analysis reveals direction dependent Far Red distribution. For each predefined azimuth *θ* and polar angle *φ*, a line from the MCTS center point to the surface was defined (a). The line voxel indices in the line was used to acquire the line histogram in the Far Red raw image data (b) and segmented Far Red volume (c), Figure S3: Center to surface line histogram analysis. Using the line data from the segmented Far Red volume, the MCTS radius was defined as the full line vector length. The total amount of Far Red positive voxels was used to calculate the local shell thickness and the MCTS core radius was defined as the distance between the MCTS center and the first non-zero element in the line data vector. The first non-zero element was also used to define the MCTS core mask.
