# Supplementary material for: Ultrasound-Based Scaffold-Free Core-Shell Multicellular Tumor Spheroid Formation
Source: Micromachines (Basel). 2021 Mar 20;12(3):329. doi: 10.3390/mi12030329 (PMC8003921; doi:10.3390/mi12030329)
Supplement: Supplementary file 1 [file micromachines-12-00329-s001.pdf]

Supplementary Information: Ultrasound-based scaffold-free core-shell multicellular tumor spheroid formation

Karl Olofsson[1], Valentina Carannante[2], Madoka Takai[3], Björn Önfelt[1,2] and Martin Wiklund[1]*

[1]*Department of Applied Physics, Science for Life Laboratory, KTH Royal Institute of Technology, SWEDEN*

[2]*Department of Microbiology, Tumor and Cell Biology, Science for Life Laboratory, Karolinska Institutet, SWEDEN*

[3]*Department of Bioengineering, University of Tokyo, JAPAN*

*Correspondence: martin.wiklund@biox.kth.se

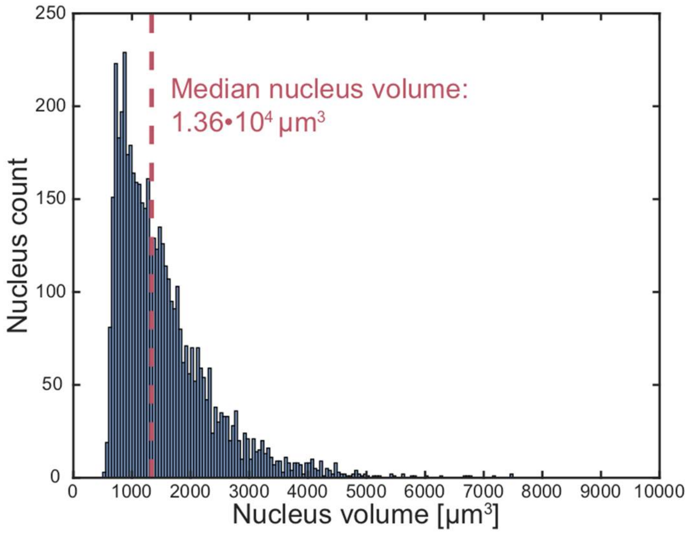

Figure S1: Median nucleus volume acquisition. Histogram shows the nucleus volume after watershed segmentation. The red dashed line indicates the median nucleus volume used to calculate the number of nuclei.

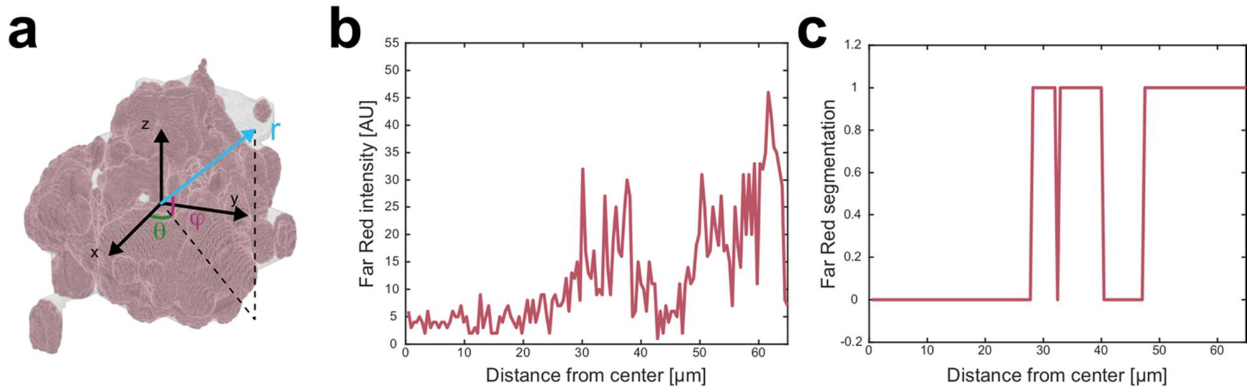

Figure S2: Center to surface line analysis reveals direction dependent Far Red distribution. For each predefined azimuth θ and polar angle φ, a line from the MCTS center point to the surface was defined **(a)**. The line voxel indices in the line was used to acquire the line histogram in the Far Red raw image data **(b)** and segmented Far Red volume **(c)**.

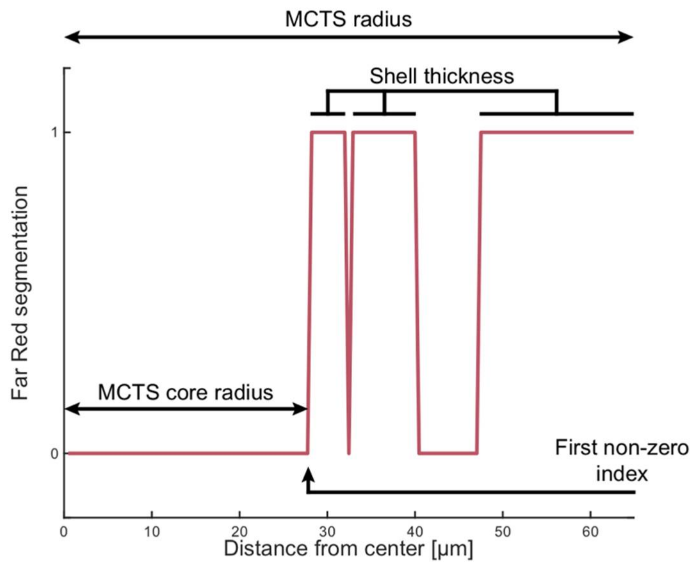

Figure S3: Center to surface line histogram analysis. Using the line data from the segmented Far Red volume, the MCTS radius was defined as the full line vector length. The total amount of Far Red positive voxels was used to calculate the local shell thickness and the MCTS core radius was defined as the distance between the MCTS center and the first non-zero element in the line data vector. The first non-zero element was also used to define the MCTS core mask.
